# Incidence of primary graft dysfunction after lung transplantation is altered by timing of allograft implantation

**DOI:** 10.1136/thoraxjnl-2018-212021

**Published:** 2018-10-09

**Authors:** Peter S Cunningham, Robert Maidstone, Hannah J Durrington, Rajamayier V Venkateswaran, Marcelo Cypel, Shaf Keshavjee, Julie E Gibbs, Andrew S Loudon, Chung-Wai Chow, David W Ray, John F Blaikley

**Affiliations:** 1 Faculty of Biology, Medicine and Health, Manchester Academic Health Sciences Centre, The University of Manchester, Manchester, UK; 2 Department of Respiratory Medicine, Manchester University NHS Foundation Trust, Manchester, UK; 3 Department of Cardiothoracic Surgery, Manchester University NHS Foundation Trust, Manchester, UK; 4 The Toronto Lung Transplant Program, Toronto General Hospital, University Health Network, University of Toronto, Toronto, Ontario, Canada; 5 NIHR Oxford Biomedical Research Centre, John Radcliffe Hospital, Oxford, UK; 6 Oxford Centre for Diabetes, Endocrinology and Metabolism, University of Oxford, Oxford, UK

**Keywords:** lung transplantation, macrophage biology

## Abstract

The importance of circadian factors in managing patients is poorly understood. We present two retrospective cohort studies showing that lungs reperfused between 4 and 8 AM have a higher incidence (OR 1.12; 95% CI 1.03 to 1.21; p=0.01) of primary graft dysfunction (PGD) in the first 72 hours after transplantation. Cooling of the donor lung, occurring during organ preservation, shifts the donor circadian clock causing desynchrony with the recipient. The clock protein REV-ERBα directly regulates PGD biomarkers explaining this circadian regulation while also allowing them to be manipulated with synthetic REV-ERB ligands.

## Introduction

Primary graft dysfunction (PGD) is an early complication of lung transplantation occurring within 72 hours of organ implantation, resulting in significantly increased morbidity and mortality.^1^ It is thought to result from ischaemia/reperfusion injury, partially mediated by myelomonocytic cell activation of lymphocytes and neutrophils.^2^ Although risk factors for PGD have been extensively studied, the effect of operation time on PGD incidence has yet to be investigated despite murine models suggesting ischaemia/reperfusion injury is under circadian control after renal transplantation.^3^

One mechanism that could link PGD incidence to operation time is temporal gating of inflammatory responses via the circadian clock.^4^ This clock is an evolutionarily conserved protein network which oscillates with a 24-hour period. The circadian clock is aligned to the external environment through environmental zeitgebers, for example, temperature, which shifts the clock’s phase.

To investigate the impact of circadian clock phase on lung transplantation, we studied whether the incidence of PGD is affected by the time of day organ reperfusion occurs. Since circadian oscillations continue *ex vivo*,^5^ we hypothesised that sudden temperature changes during organ preservation may shift the donor’s clock, resulting in donor recipient circadian desynchrony and an increased incidence of PGD.

## Methods

Two retrospective cohort studies were analysed. The initial retrospective cohort study was used to define whether a high-risk window for organ implantation existed. The subsequent larger retrospective validation cohort was used to confirm these results at a different transplant centre. For this study, patients were included if they received a lung transplant between 2004 and 2012, were >18 years of age and did not have any significant intraoperative complications. The primary endpoint was defined before data were collected as the development of PGD grade 2/3.

PER2::luc reporter mice were used to identify the effects of organ preservation on circadian clock oscillations. Peritoneal exudate cells were obtained from mice as previously described.^4^ Alveolar macrophages were obtained from lung transplant recipient bronchoalveolar lavage fluid (BAL) fluid during routine surveillance bronchoscopy.

Statistical analysis and further methodological details are in the online supplementary data.

10.1136/thoraxjnl-2018-212021.supp1 Supplementary data

## Results

**Figure 1:**
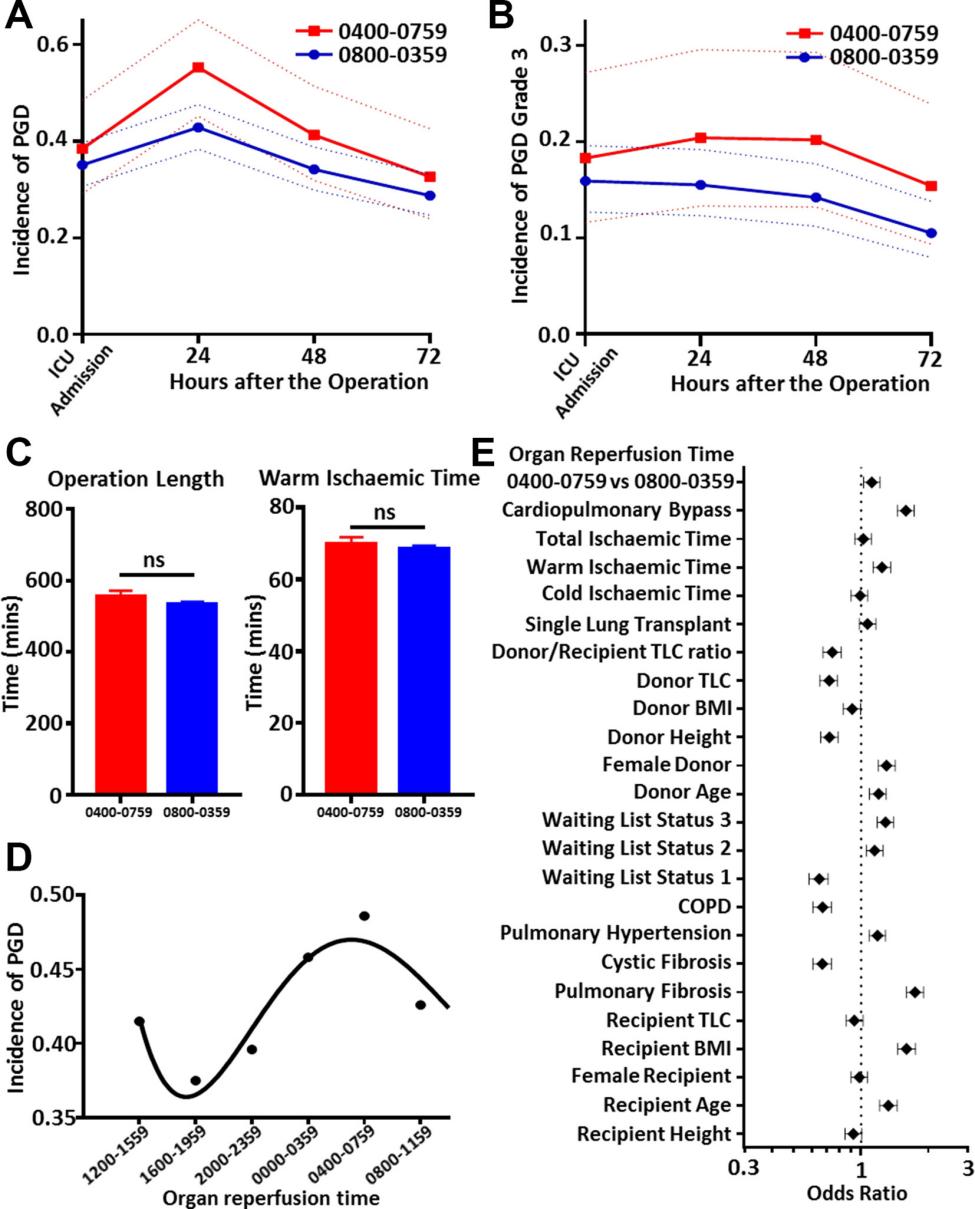
Lungs transplanted between 4 and 8 AM have a higher incidence of PGD. Results from a retrospective cohort study showed that lungs reperfused between 4 and 8 AM had a higher incidence of PGD grades 2 and 3 after transplantation (p=0.01, univariate logistic regression) (A). A similar effect was seen for the severest form of PGD, grade 3, however, this was not significant (p=0.15, univariate logistic regression) (B). No difference was seen in surrogate markers of human performance between the two time points (C). Analysis of double lung transplant recipients from the same cohort controlling for circadian factors revealed that PGD incidence oscillated in a sinusoidal manner 24 hours after the operation (p=0.03, EU circwave) (D). A number of other covariates were also measured in our cohort (E), the effect of reperfusion time was still seen after controlling for these in the multivariate logistic regression model. BMI, body mass index; COPD, chronic obstructive pulmonary disease; PGD, primary graft dysfunction; TLC, total lung capacity.

**Figure 2:**
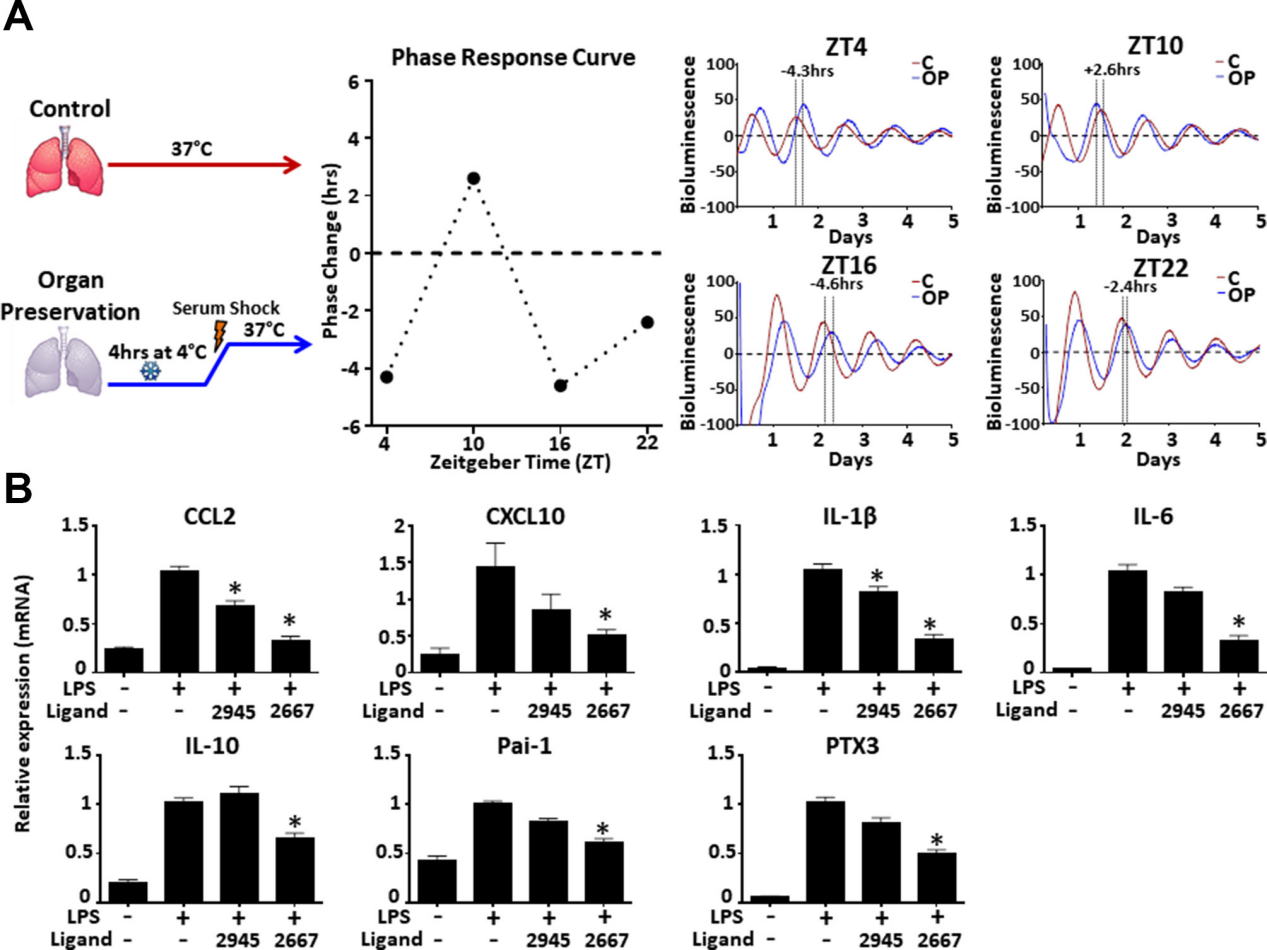
PGD biomarkers are direct targets of the cellular clock whose phase is altered by organ preservation. Lungs were either kept at 37°C or rapidly cooled and rewarmed mimicking organ preservation. Assessment of circadian phase revealed that circadian oscillations were altered in lungs exposed to temperature alterations (A). Alveolar macrophages from transplant recipients were exposed to two synthetic compounds acting as agonists for the key clock protein, REV-ERBα. 2667 repressed all seven PGD biomarkers (*p<0.05, t-test) (B). PGD, primary graft dysfunction.

The pilot study (n=25) suggested that lungs reperfused between 0400–0759 have a higher incidence of PGD (online supplementary figure 1A). To confirm these findings a larger retrospective cohort (n=563) study, at a different transplant centre, was performed. This showed that lungs reperfused during the high-risk window had a small but significant (OR 1.12, 95% CI 1.03 to 1.21; p=0.01 univariate binary logistic regression figure 1A,E and online supplementary tables 1 and 2; OR 1.299, 95% CI 1.004 to 1.681, p=0.046 multivariable binomial logistic regression; online supplementary table 3) increase in incidence of PGD. Secondary analysis using PGD grade 3 incidence found a similar but non-significant (p=0.15) (figure 1B) difference. One explanation for these findings could be human operator fatigue, however no difference was observed for surrogate markers of human performance, for example, warm ischaemic time or operation length (figure 1C). We therefore hypothesised that this could result from internal desynchrony between donor and recipient as a result of organ preservation. This was supported by a subgroup analysis, controlling for type of operation and factors that could affect circadian disruption. The subgroup included all double lung transplant recipients who were not relatively contraindicated by weight or age according to ISHLT criteria.^6^ This showed that early (24 hours) PGD oscillated in a sinusoidal manner (figure 1D, online supplementary figure 1b) peaking at the same time as the original cohort (online supplementary figure 1c). A mouse model was then used to establish whether temperature shifts, a standard part of lung procurement, altered circadian clock oscillations. Lungs from PER2::luc mice, which allow real-time recording of circadian oscillations,^7^ were exposed to temperature shifts mimicking organ procurement or kept at 37°C. A phase advance or delay was seen for lungs exposed to temperature shifts (figure 2A), supporting our hypothesis that circadian desynchrony may result from the organ preservation and implantation protocols in routine clinical use.

The clock protein, REV-ERBα, modifies ischaemic injury after cardiac surgery and its function can be modified by synthetic ligands. PGD biomarker gene expression^8^ (CCL2, CXCL10, IL-1β, IL-6, IL-10, Pai-1, PTX3) was therefore examined in human monocyte-derived macrophages by a gene array.^4^ These were all repressed by the synthetic REV-ERBα ligand (GSK4112). Six out of the seven biomarkers showed higher expression in LPS-stimulated peritoneal macrophage cells from REV-ERBα knockout mice confirming that they are indeed genuine REV-ERBα targets (online supplementary figure 1D). The REV-ERBα ligands were also tested on alveolar macrophages from lung transplant recipients. One of the ligands repressed two out of the seven biomarkers, while the other ligand repressed all seven (figure 2B).

## Discussion

As with many clinical studies, this study can only show association rather than causation. In line with another study,^9^ we believe that human factors cannot be the sole explanation as there were no differences in surrogate markers of human performance. It is currently impossible to record real-time clock oscillations in humans, therefore we used a mouse model to show that organ preservation causes marked circadian clock phase shifts, resulting in internal desynchrony between the donor organ and recipient. Although this study does not exclude that other circadian output pathways may be involved, the importance of REV-ERBα in conveying circadian signals to inflammatory gene expression in macrophages is unquestioned.^10^ In our study, REV-ERBα null mice were used to confirm that the PGD biomarker genes were genuine REV-ERBα targets. Unexpectedly, *PTX3* gene expression was repressed both in the REV-ERBα null mice and by the REV-ERB ligands in human cells indicating a more complex mechanism of REV-ERB regulation for this gene.

The following limitations should be noted. Since both clinical studies were retrospective, the caveats that apply to all retrospective studies should be applied. The use of ORs can inflate associations if the study cohort varies in size during the investigation period however this did not happen in our study. Finally to establish causation, a lung transplant model could be used to investigate circadian regulation of PGD establishing that circadian control of ischaemia/reperfusion injury extends beyond the kidney.^3^

This is the first study showing that circadian factors are important in transplant surgery. Although the effect was small, circadian factors should be taken into account in future PGD studies; furthermore ligands targeting REV-ERBα show promise as novel therapeutic compounds for PGD.
