# Supplementary material for: Incidence of primary graft dysfunction after lung transplantation is altered by timing of allograft implantation
Source: Thorax. 2018 Oct 9;74(4):413–6. doi: 10.1136/thoraxjnl-2018-212021 (PMC6484691; doi:10.1136/thoraxjnl-2018-212021)
Supplement: Supplementary data [file thoraxjnl-2018-212021supp001.pdf]

Supplementary Results

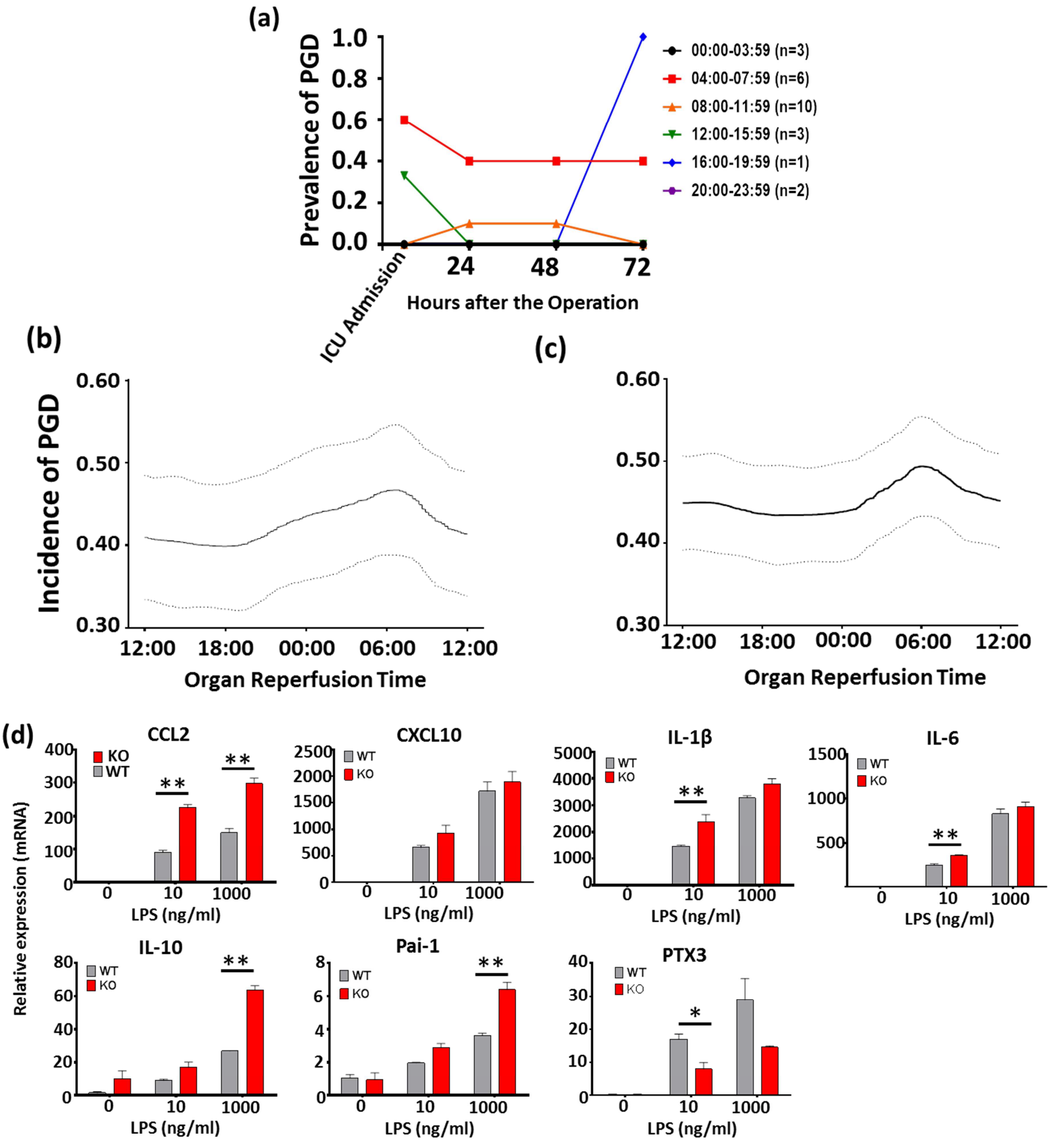

Suppl. Figure 1: A pilot study was performed to see whether circadian factors influenced the incidence of primary graft dysfunction. Organs reperfused between 0400-0759 had a higher incidence of primary graft dysfunction **(a).** LOESS analysis of the sub-cohort, defined according to ISHLT criteria, suggested that PGD incidence oscillated in a smoother circadian manner **(b)** compared to the main cohort **(c)** (±95% CI shown). To investigate if REV-ERBα regulated PGD biomarkers, peritoneal exudate cells from REV-ERBα knockout mice were stimulated with LPS. Six out of the seven biomarkers had higher levels of expression after LPS stimulation (*=p<0.05, **=p<0.01, t test)**(d).**

Suppl. Table 1: **Cohort demographics**. Time of organ re-perfusion was documented in 563 recipients. Results are expressed as actual number (percentage) or as mean (±SD). Statistical analysis was conducted using independent t test or Chi-square where appropriate.

**Organ reperfusion time**		04:00-07:59	08:00-03:59	p-value
Number		104	459	
**Donor variables**				
Females		50 (48.1%)	242 (52.7%)	0.455
Age		43.68 (±17.26)	44.60 (±17.33)	0.626
BMI		25.70 (±4.41)	25.73 (±5.55)	0.950
Predicted Total Lung Capacity		6.48 (±1.15)	6.30 (±1.19)	0.168
Smoker		57 (58.8%)	227 (52.1%)	0.279
**Recipient variables**				
Females		44 (42.3%)	192 (41.8%)	1.000
Age		50.53 (±14.46)	51.48 (±14.54)	0.547
BMI		24.16 (±4.11)	23.34 (±4.21)	0.070
Underlying Lung Disease	COPD and alpha-1-antitrypsin deficiency	27 (26.0%)	130 (28.3%)	0.359
	Cystic fibrosis	19 (18.3%)	110 (24.0%)	
	Primary Pulmonary Hypertension	7 (6.7%)	15 (3.3%)	
	Pulmonary fibrosis	45 (43.3%)	180 (39.2%)	
	Other	6 (5.8%)	24 (5.2%)	
Status at Transplant	1	25 (24.0%)	123 (26.9%)	0.826
	2	56 (53.8%)	241 (52.6%)	
	3	23 (22.1%)	94 (20.5%)	
**Operative variables**				
Bilateral Transplant		85 (81.7%)	402 (87.6%)	0.156
Best PaO_2 of donor Lung		444.00 (±88.12)	447.70 (±86.54)	0.703
Donor/Recipient TLC ratio		1.02 (±0.17)	1.01 (±0.17)	0.406
Cold Ischaemic Time (min)		281.68 (±107.95)	315.11 (±102.17)	0.003
Warm Ischaemic Time (min)		69.82 (±19.01)	68.47 (±19.63)	0.529
Operation length (min)		555.84 (±165.08)	534.29 (±131.62)	0.157

Suppl. Table 2: Univariate model. Predictors for PGD were analysed using univariate binomial logistic regression. Significant ($p<0.05$) variables are highlighted in bold and these were fed into the multivariable binomial logistic regression

Predictor	Odds Ratio (standardised)	OR Confidence Interval		p-value
		2.5%	97.5%	
Recipient Height	0.924	0.847	1.007	0.070
Female Recipient	0.982	0.902	1.070	0.683
**Recipient Age**	**1.325**	**1.212**	**1.452**	**<0.001**
**Recipient BMI**	**1.600**	**1.461**	**1.7531**	**<0.001**
**Donor Age**	**1.200**	**1.090**	**1.297**	**<0.001**
**Female Donor**	**1.300**	**1.195**	**1.422**	**<0.001**
**Donor Height**	**0.721**	**0.659**	**0.788**	**<0.001**
**Donor BMI**	**0.913**	**0.834**	**0.996**	**0.043**
**Donor TLC**	**0.720**	**0.654**	**0.783**	**<0.001**
Cold Ischaemic Time	0.99	0.902	1.071	0.700
**Warm Ischaemic Time**	**1.240**	**1.132**	**1.357**	**<0.001**
Total Ischaemic Time	1.022	0.938	1.114	0.620
**Cardiopulmonary Bypass**	**1.586**	**1.455**	**1.730**	**<0.001**
**Donor Recipient Lung Ratio**	**0.742**	**0.675**	**0.814**	**<0.001**
**Lung Reperfusion Time (04:00-07:59)**	**1.116**	**1.025**	**1.213**	**0.011**
**Waiting list status 1**	**0.650**	**0.585**	**0.710**	**<0.001**
**Waiting list status 2**	**1.150**	**1.055**	**1.255**	**0.002**
**Waiting list status 3**	**1.286**	**1.182**	**1.398**	**<0.001**
**COPD**	**0.672**	**0.610**	**0.737**	**<0.001**
**Pulmonary Fibrosis**	**1.746**	**1.600**	**1.906**	**<0.001**
**Cystic Fibrosis**	**0.670**	**0.610**	**0.738**	**<0.001**
**Pulmonary Hypertension**	**1.183**	**1.088**	**1.287**	**<0.001**
Single Lung Transplant	1.069	0.982	1.163	0.123
Recipient TLC	0.933	0.857	1.02	0.1168

Suppl. Table 3: Multivariate model. Predictors for PGD were analysed using multivariate binary logistic regression using both forwards and backwards selection. Significant ($p<0.05$) variables for the final model are shown in the table along with the confidence intervals.

**Predictor**	**Odds Ratio (unstandardised)**	**OR Confidence Interval**		**p-value**
		**2.5%**	**97.5%**	
Lung Reperfusion Time (04:00-07:59)	1.299	1.003	1.681	0.046
Cystic Fibrosis	0.492	0.305	0.796	0.004
COPD	0.538	0.339	0.857	0.009
Cardiopulmonary Bypass	1.857	1.504	2.293	<0.001
Waiting List Status 2	2.032	1.529	2.715	<0.001
Waiting List Status 3	2.454	1.755	3.446	<0.001
Recipient BMI	1.068	1.039	1.099	<0.001
Donor Age	1.007	1.001	1.013	0.016

Supplementary Methods

Patient cohort in the U.K. (pilot study): To establish the time bins and comparisons for a larger retrospective study a preliminary study was performed at a different transplant centre. Patients were included if they received their transplant operation between 2010-12 (n=25) and the organ reperfusion time could be identified from the notes. These patients were split into six four-hour groups by the time the first lung was reperfused during transplantation. PGD grades were then assigned after reviewing their radiology, case notes and blood gases at four established time-points according to ISHLT criteria.

Patient Cohort in Canada: Patients were identified from a local database used to record transplant activity at Toronto General Hospital, Toronto Canada. Patients were included in the study if they received a single or double lung transplant. Recipients were excluded from the study if they were under 18 at time of transplantation, had a significant intra-operative event that was recorded on the database e.g. bleeding, had a previous lung transplant or the donor lungs had been examined using ex-vivo lung perfusion. All inclusion and exclusion criteria were assigned before analysis began. Lung allocation score (LAS) is assigned to patients in the Toronto transplant program by a multi-disciplinary team assessment. A score of 1 is assigned where the recipient's condition is stable or deteriorating slowly, a score of 2 is assigned where the recipient is deteriorating rapidly, a score of 3 is assigned when the patient has been admitted to intensive care. PGD score was used from the same database, if this was unclear or had not been assigned then the patient's record was examined directly from the electronic record. Organ reperfusion time was defined as the earliest time-point after organ retrieval that the donor lung(s) were reperfused with recipient blood, this was usually recorded on the anesthetic chart. Patients were split into two groups according to organ reperfusion time defined by the pilots study performed at a Transplant center in the U.K. Primary outcome was defined as the difference in incidence for primary graft dysfunction (grades 2&3) recorded at four time-points after transplantation for patients whose organs were reperfused during the high risk window (0400-0759) compared to organs reperfused between 0800-0359. Secondary analysis repeated the analysis for primary graft dysfunction grade 3 alone, and also for a sub-cohort of double lung transplant recipients that were not relatively contra-indicated due to BMI or age. The latter analysis was performed to control for the effects of operation length, age and obesity on circadian rhythms.

Ethical Approval: Ethical approvals for both human and animal studies were obtained.

Statistical Analysis: Statistical analysis was performed using R. Univariate binomial regression models were fitted to determine the significance of individual variables on PGD incidence as well as to generate odds ratios (fig 1e, suppl. table 2). For the multivariate analysis a binomial regression model was created using bidirectional elimination. Co-linearity of the covariates was evaluated using the generalised variance inflation factor, scaled by the degrees of freedom of the variables[1]. Possible variables were initially selected using biological plausibility established from previous PGD studies or they had a significance of less than 0.2 in the univariate analysis. The Akaike information criterion (AIC) was used to determine which variables were removed or reintroduced at each stage of the bidirectional elimination. Covariates were added or removed from the model if doing so would reduce the AIC. Sub cohort analysis was performed using EU circwave [2] on a defined cohort of patients that were not relatively contra-indicated as a result of age/ obesity to lung transplant according to the ISHLT criteria[3]. Initially incidence of PGD (grades 2/3) 24 hours after surgery was calculated for each of the six four-hour bins based on when the first lung was re-perfused in the recipient. These were then plotted against time of organ reperfusion following which regression was performed using EU circwave. The primary analysis was done using all timepoints after surgery were PGD was measured. The multivariate model included the following co-variates Reperfusion Time, Time since Operation, Recipient Age, Disease Group, Cardiopulmory Bypass, Waiting List Status, BMI and Donor Age. LOESS regression was implemented using R to smooth the PGD incidence data taking into account the individual patient reperfusion times (suppl. fig. 1b). The span parameter for the LOESS was chosen using the elbow method on the residual sum of squares.

Generation of monocyte derived macrophages for microarray analysis: 50 mls of blood was taken from six human volunteers. These volunteers did not have any active health complaints, had not taken any medication for 6 weeks prior to sampling and were not involved in any other clinical studies. The blood was diluted 1:2 with RPMI 1640 (Gibco) and layered over ficoll-paque. This solution was then spun for 30 minutes at 4°C after which the interphase layer was taken off. This was then centrifuged for 5minutes at 300g and the pellet was re-suspended in RPMI 1640 (supplemented with penicillin/ streptomycin and 10%FBS) at 5x10^{6} cells/ml. All the cells were then plated out into 24 well plates and left to adhere for 1 hour at 37°C, following which each well was washed three times and the plates were incubated overnight. Media was changed in all the wells the following morning and also every alternate day until the cells had become monocyte derived macrophages after 10 days. The cells were then stimulated with LPS (10µg/ml) in the presence or absence of GSK 4112 (10µM). After 6 hours the cells were lysed using a RNAeasy kit (Qiagen) and analysed on a microarray platform using the affymetrix gene chip U133 plus 2.0 array and normalized using the RMA algorithm. Significant gene changes were defined by an average fold

change of ±2 over all the six patients. Gene changes were separated into repressed genes (n=120) and induced genes (n=22). String analysis, DAVID and Ingenuity analysis was used on the dataset to infer biologically relevant pathways.

Peritoneal exudate Cells: 10mls of phosphate buffered saline was injected into the peritoneal cavity and recovered. The fluid was spun down at 500xg for 8 minutes, after which cells were resuspended in RMP 1640 (10% FBS, penicillin and streptomycin) at a seeding density $1x10^6$ cells/ml. 0.5mls was added to a 24 well plate, after which the cells were left to adhere and washed three times the following morning after which the cells were stimulated with LPS for six hours and then lysed for RNA extraction.

Alveolar macrophages form BAL: Bronchoalveolar lavage was performed as part of routine care for lung transplant recipients. Any fluid not required for clinical analysis was given to researchers and used in the study. The BAL fluid was passed through a 100μM strainer, spun down at 500xg for 5 minutes and resuspended (RPMI 1640 with 10% FBS, penicillin and streptomycin) at $1.25x10^6$ cells per ml. This cellular suspension was seeded out into a 96 well plate and left for an hour at 37°C. Each well was then washed three times following which the plate was left in the incubator overnight. The following morning the wells were washed once more before the experiment began.

Simulation of organ preservation: Mice kept in L::D and D::L were sacrificed at two different time points. Lungs were either harvested and analysed directly or perfused with ice cold PBS and stored for four hours on ice prior to exposure to 50% serum for 2 hours at 37°C followed by experimental analysis. Small slices of lung from mice on a PER2::luc background were placed in 35mm dish and cultured in luciferin containing media[4]. 24 lung slices from 8 mice were used to create the data in fig 2a with traces showing the average for three lung slices. Differences for Per2::Luc phasing were calculated by calculating the different times between the 2nd peaks from the lumicorder trace.

qPCR:

RNA was extracted using the ReliaPrep system (Promega). qPCR was performed using Power SYBR Green (Thermo) and QuantiTect primer assays (Qiagen).

Gene Array: The RNA was amplified, reverse transcribed and then hybridised onto an Affymetrix U133 plus 2.0 chip. Data was normalised using the RMA algorithm[5] and log (2-based) transformation was performed. Paired t-test with limma correction was used to identify those genes significantly regulated by GSK 4112. DAVID (v. 6.7)[6 7] was used to analyse the function of the significantly

regulated genes. Only genes that were differentially expression >2-fold were included. Results have been uploaded to array express with the id E-MTAB-6478

Animals

All animals were maintained in 12h:12h light:dark (LD) with food and water supplied ad libitum. Male C57BL/6J mice were purchased from Charles River (UK). mPER2::luc transgenic mice and global *Reverbα* KO mice were previously described.

Chemicals

Lipopolysaccharide expressed by the *E.Coli* strain 026:B6 was obtained from Sigma-Aldrich Corp (Missouri USA). The REVERB agonists, GSK 4112, 2667 and 2945 have been previously described [8 9] and were a kind gift from GSK.

References

1. Fox J, Monette G. Generalized Collinearity Diagnostics. *Journal of the American Statistical Association* 1992;87(417):178-83.
2. Batista RT, Ramirez DB, Santos RD, del Rosario MC, Mendoza ER. EUCLIS--an information system for circadian systems biology. *IET.Syst.Biol.* 2007;1(5):266-73.
3. Weill D, Benden C, Corris PA, Dark JH, Davis RD, Keshavjee S, et al. A consensus document for the selection of lung transplant candidates: 2014--an update from the Pulmonary Transplantation Council of the International Society for Heart and Lung Transplantation. *J Heart Lung Transplant* 2015;34(1):1-15.
4. Yamazaki S, Takahashi JS. Real-time luminescence reporting of circadian gene expression in mammals. *Methods in enzymology* 2005;393:288-301.
5. Irizarry RA, Hobbs B, Collin F, Beazer-Barclay YD, Antonellis KJ, Scherf U, et al. Exploration, normalization, and summaries of high density oligonucleotide array probe level data. *Biostatistics.* 2003;4(2):249-64.
6. Dennis G, Jr., Sherman BT, Hosack DA, Yang J, Gao W, Lane HC, et al. DAVID: Database for Annotation, Visualization, and Integrated Discovery. *Genome Biol.* 2003;4(5):3.
7. Huang dW, Sherman BT, Lempicki RA. Systematic and integrative analysis of large gene lists using DAVID bioinformatics resources. *Nat Protoc.* 2009;4(1):44-57.
8. Grant D, Yin L, Collins JL, Parks DJ, Orband-Miller LA, Wisely GB, et al. GSK4112, a Small Molecule Chemical Probe for the Cell Biology of the Nuclear Heme Receptor Rev-erbalpha. *ACS Chem.Biol.* 2010.
9. Trump RP, Bresciani S, Cooper AW, Tellam JP, Wojno J, Blaikley J, et al. Optimized chemical probes for REV-ERBalpha. *J.Med.Chem.* 2013;56(11):4729-37.
